# Notes and Notices

**Published:** 1891-04

**Authors:** 


### NOTES AND NOTICES.

THE INDIANA INSTITUTE OF HOMŒOPATHY will hold its quarto-centenary meeting at Indianapolis, May 13th and 14th, 1891.

From a small and weak body, for years struggling for existence through the trials and self-denials of its early defenders, yet full of enthusiasm and hope, the State Society of the Homœopathic Physicians of Indiana has, with the slow growth that betokens ultimate solidity and perpetuity, become large and powerful. This truth was most clearly proven at last year's meeting, when the unprecedented number (for any State Society) of forty-one acceptable candidates for admission to it presented themselves and were elected members, and when the interest was so great and sustained that an evening session had to be held on the last day (for the first time in its history), in order to hear and discuss all the interesting and valuable papers awaiting presentation.

This year new features will be introduced, the chief of these being that (as the Institute will this year publish its transactions in pamphlet form, for the first time in its history) all the discussions will be stenographically reported by an experienced medical reporter.

Non-members to whom this circular may come hardly need reminding that their names cannot appear in this volume. The requirements constituting eligibility to membership of licensed physicians are simply the subscribing to the belief in Similia and the payment of two dollars membership fee and the annual dues of two dollars, the payment of which entitles one to the fine large steel-engraved certificate of membership and a copy of the transactions of that year, to say nothing of the privileges of association.

The Secretary must early have the titles of all papers to be read in order to make up the programme, and they must reach him not later than the end of April. Address, WM. B. CLARKE, M. D., *Secretary Indiana Institute of Homœopathy*, 7 Mansur Block, Indianapolis, Ind.

THE BOSTON HAHNEMANNIAN ASSOCIATION.—The pure homœopathists of Boston have formed themselves into a society for the maintenance of the *one* law of cure, and have taken the name of *The Boston Hahnemannian Association.*

They have adopted a platform of principles, a Constitution and By-Laws very similar to the International Hahnemannian Association. We give herewith their DECLARATION OF PRINCIPLES:

The following resolutions express the sentiments and represent the practice of the members of the Boston Hahnemannian Association.

WHEREAS, We believe the law of similars to be the law of cure; we believe a proper knowledge of the curative power of medicines to be derived from provings made upon healthy persons; we believe Hahnemann's *Organon of the Healing Art* to be the true guide in therapeutics; that the totality of the symptoms forms the only basis for the selection of the remedy, and that the best results are attained by the use of the minimum dose of the single remedy in a potentiated form; therefore, be it

*Resolved*, That we adopt the name "Hahnemannian Homœopathists," in contradistinction to that of "Homœopathists," which has been and is misappropriated by those who claim to practice Homœopathy, but who do not comply with the conditions of the law as deduced by Samuel Hahnemann.

*Resolved*, That either the alternation or combination of remedies in prescribing is non-homœopathic.

*Resolved*, That the use of local applications, unless homœopathically indicated, is non-homœopathic.

*Resolved*, That mechanical appliances are admissible only when mechanical conditions are to be overcome.

*Resolved*, That we deprecate any practice which tends to the suppression of symptoms, inasmuch as it injures the patient and renders difficult the selection of the specific remedy.

International Hahnemannian Association.—The next meeting will be held at the Spring House, Richfield Springs, N. Y., June 23d, 24th, 25th, and 26th, 1891; hotel rates, two dollars and fifty cents per day. It is important that there should be a large attendance, as action is to be taken upon the revised Constitution and By-Laws. If it is possible to be present, it is the duty of every member to come, to assist in upholding the Homœopathy of Hahnemann, and to protest against the practices contrary to the homœopathic law, which, under the guise of Homœopathy, are daily increasing. This cannot be done by remaining at home and trusting to others to carry on the work, but every one should contribute a share to the success of the meeting by writing a paper and by being present. Address, S. A. Kimball, M. D., *Secretary I. H. A.*, 124 Commonwealth Avenue, Boston, Mass.

Children's Homœopathic Hospital, 914 North Broad Street, Philadelphia.—The competitive examination for resident and junior resident physicians will be held at the hospital on Saturday, April 4th. Applications should be sent to the President of the Medical Board.

The Hahnemannian's Analysis Sheet.—The author, Dr. M. A. A. Wolff, Gainesville, Texas, announces that he has caused to be printed a cheaper edition, without directions, which he will sell at one dollar a hundred copies, one copy containing directions being added to insure correct use. He will also sell the entire copyright, plates, etc., at a low price.

Dr. A. B. Norton, 152 West 34th Street, New York, announces that he has succeeded to the practice of his brother, the late Dr. George S. Norton, as an exclusive specialist upon the eye and ear.

Fun for Doctors.—Widower.—"Doctor, your bill is something fearful. After you have doctored my wife to death, you expect me to pay you an enormous bill."

Doctor.—"That's just what I expected you to say. Such a thing as gratitude no longer exists in this world."—*Texas Siftings*.

